# Accessory Central Cusp in the Maxillary Second Primary Molars: A Rare Entity among the Rare

**DOI:** 10.5005/jp-journals-10005-1265

**Published:** 2015-02-09

**Authors:** Biswaroop Chandra, Monalisa Das

**Affiliations:** Senior Lecturer, Department of Pedodontics and Preventive Dentistry, Guru Nanak Institute of Dental Science and Research, Kolkata West Bengal, India; Postgraduate Trainee, Department of Pedodontics and Preventive Dentistry, Guru Nanak Institute of Dental Science and Research, Kolkata West Bengal, India

**Keywords:** Accessory cusp, Central cusp, Dens evaginatus.

## Abstract

Central accessory cusp in primary molars is an extremely rare condition which is due to abnormal proliferation and folding of inner enamel epithelium during morphodifferentiation stage of tooth development. The extension of the pulp in the cuspal area is often the reason for early involvement of pulp by the caries process. Therefore, early diagnosis and treatment planning is necessary to maintain the integrity of primary dental arch. This article reports such a case of central accessory cusps involving maxillary second primary molars.

**How to cite this article:** Chandra B, Das M. Accessory Central Cusp in the Maxillary Second Primary Molars: A Rare Entity among the Rare. Int J Clin Pediatr Dent 2014;7(3):202-205.

## INTRODUCTION

In this world, every normal creation has a possibility to present with abnormality to some extent, called rare and these rare events or things are always exceptional but special. After a long developmental process including histodifferentiation and morphodifferentiation a tooth gets its adult form. Any change or deviation of this pattern of development may alter the normal consequences. Both primary and permanent dentition may show structural variations involving crown and/or root of the affected teeth.

Accessory cusps are occasional to rare. They present as minor variations to gross defects of the cusps. The cusp of Carrabelli of the molars is the most commonly reported accessory cusp, whereas talon's cusp in the incisors and leong's tubercle in premolars are not so common incidences. These morphological variations can be seen both in primary and in permanent dentitions.^1^ The frequency of occurrence of these variations differ depending on their type; Talon's cusp (between 1 and 7.7%) and Carabelli's cusp (68%) and Leong's tubercle (8%).^2^

The ‘central cusp’ is actually an occlusal supernumerary cusp, situated between the buccal and the lingual cusp tips of the premolars and molars and on the lingual surface of the incisors and canine. Leigh in 1975, first reported the central cusp as an enamel tubercle on the maxillary right third molar of an Eskimo's skull.^3^ Several authors subsequently described the central cusp in their literature,^4,5^ in different teeth with different forms. Lau,^4^ Merrill^6^ and Schulze^7^ suggested different forms of central cusps and grouped them on the basis of their location and shapes with special regard to the premolars.

It was assumed in the past that the formation of accessory cusp occurs due to over activity of the dental lamina. But now it is believed that the PAX and MSX genes are responsible for the abnormal cuspal morphology of the teeth.^8^ During bell stage of tooth development, the altered proliferation and folding of a portion of the inner enamel epithelium and subjacent ectomesenchymal cells of the dental papilla forms a supplemental solid elevation on the crown surface. The embryological evidence suggests that tooth morphogenesis is characterized by transient signalling centers in the epithelium. They consist of epithelial cell clusters that correspond to the initiation of the formation of individual cusps.^9,10^ These signalling centers of epithelial cells and the primary and secondary enamel knots, serve a regulatory function.^11^

The primary enamel knot regulates the advancing cuspal morphogenesis of the crown through expression of up to 20 molecules including fibroblast growth factors FGF-4 and FGF-9, transforming growth factor-b and bone morphogenic proteins BMP-2,4 and 7, etc. The mesenchy-mal BMP-4 induces expression of p21, possibly linked to the programme disappearance of the primary knot cells.^12^

Activator from the primary enamel knot regulates the expression of secondary enamel knots. The resultant cusp morphogenesis and positions appear to be determined sequentially, and cusps that form late in development, are typically small.^13^ The actual number of cusps are determined by the specific signalling molecules from the mesenchymal cells and simultaneous production of other molecular signals may induce differentiation of Hertwig's epithelial root sheath at the cervical loop.^12^

The present article reports a case of bilateral anomalous accessory central cusp of the maxillary second primary molars including the associated clinical problems.

## CASE REPORT

The incidental clinical findings of central accessory cusps were observed in a 5 years old Bengali girl who reported to the Outpatient Department of Pedodontics and Preventive Dentistry, Guru Nanak Institute of Dental Science and Research, Kolkata. Her major complain was of pain in the right and left lower back tooth region since 1 month. The pain history revealed aggravation of pain at night with an associated swelling in relation to the affected teeth. No significant relevant medical and dental histories were reported by the parents.

**Fig. 1:**
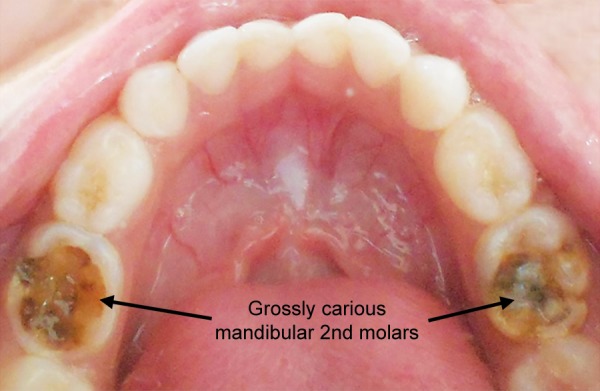
Preoperative photograph of carious lesions involving 75 and 85 opposing the accessory central cusp of maxillary 2nd molars

On clinical examination, patient had no relevant extraoral findings. The intraoral presentation revealed that the patient was in a primary dentition stage. There was presence of dental caries leading to dentoalveolar abscess with pus discharging sinus in relation to 75 and 85 (Fig. 1). The central cusps were discovered during the examination, found in both the maxillary second molars with deep pit and fssure caries (Fig. 3). No other relevant clinical findings were observed.

**Fig. 2:**
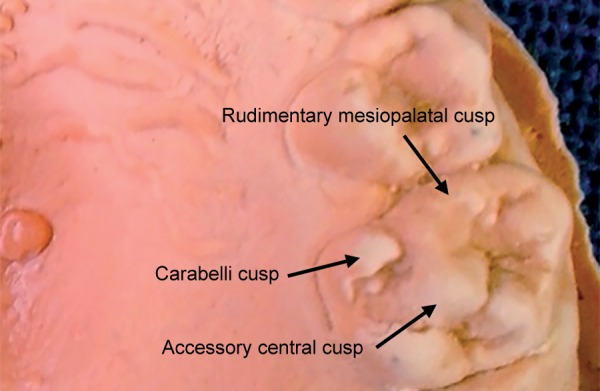
Cast model showing the occlusal anatomy of maxillary left 2nd molars

The central cusps were presented as a central projection (3 mm by 4 mm in size) on the occlusal surfaces of both the right and left maxillary second deciduous molars (Fig. 2) respectively. These cuspal projections were situated on a triangular base in the center of the oblique ridge and were identical on both sides.

The established treatment plan was pulpectomy of lower second molars followed by stainless steel crown; and light cure composite restoration of 55 and 65.

In the first phase of treatment, pulpectomy of 75 and 85 were done after draining the sinus followed by placement of stainless steel crowns in both the teeth respectively.

In the second phase of treatment, occlusal pit and fissure caries of 55, 65 were removed with the help of spoon shaped excavator (maliper) and no. 2 round diamond bur. The prepared teeth were then restored with the matched shade of packable composite (3M) preserving the cuspal morphology (Fig. 4). Next, proper postoperative instructions were given and periodic follow-up was advised to the parents. Three months and 6 months follow-up showed no eventful changes of these affected teeth.

## DISCUSSION

No two teeth are alike. Variation of teeth has been of enduring interest to the clinical practitioner and the laboratory scientist. The day-to-day variations of teeth that are seen is the norm. The odd, peculiar, and strange variations of teeth; are called anomalies.

Among different populations; the cuspal morphology of teeth varies and the accessory cusps are one group of variation which includes three patterns: cusp of Carabelli, talon's cusp and dens evaginatus. The central cusp on the occlusal surface of posterior teeth has also been given several descriptions, such as supernumerary occlusal cusp, occlusal tubercle, premolar odontome, tuberculated premolar and Leong's premolar (Scott and Turner 1997).^13^ Dens evaginatus occurs mainly in the people of Asian descent, including Japanese, Chinese, Malay, Eskimo, Thai, Filipinos and American Indians (Kocsis et al, 2002). ^3^ The reported prevalence of dens evaginatus in the Asian population is 15%; with rare occurrence in whites. Histologically, these anomalies may compose of enamel, dentine and with or without pulpal projection into them (Levitan and Himel, 2006).^14^

Though dens evaginatus is common in permanent dentition, the occurrence of central cusps in primary dentition is rare. According to the best of our knowledge, there are only two reported cases of dens evaginatus in primary second molars.^2,15^

There are various types of dens evaginatus documented by the authors. According to Schulge, five types of dens evaginatus for posterior teeth are distinguished.^7^

They can be either

1. A cone-like enlargement of the lingual cusp.
2. A tubercle on the inclined plane of the lingual cusp.
3. A cone-like enlargement of the buccal cusp.
4. A tubercle on the inclined plane of the buccal cusp, or
5. A tubercle arising from the occlusal surface obliterating the central groove.

**Fig. 3:**
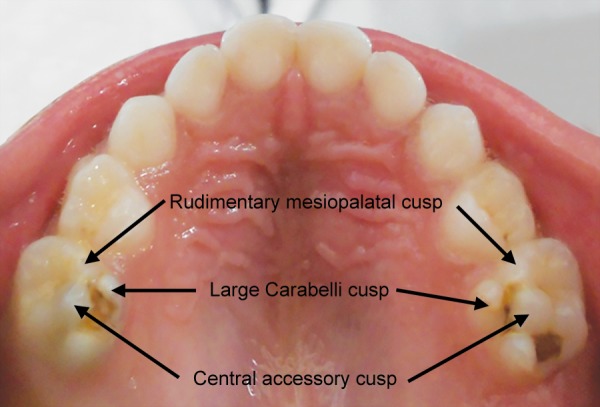
Preoperative photograph of occlusal anatomy of 55 and 85 with pit and fssure caries

**Fig. 4:**
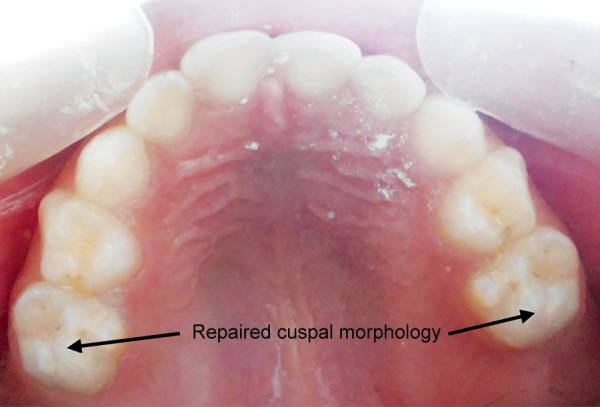
Postoperative photograph showing occlusal morphology after light cure composite restoration

According to Oehlers 1956;^5^ presence of central cusp or tubercle at the center of a tooth is often said to be due to abnormal proliferation of the epithelial fold during the morphodifferentiation phase of tooth development. The five categories are listed as follows along with their percentage of occurrence:

1. Wide pulp horns (34%)
2. Narrow pulp horns (22%)
3. Constricted pulp horns (14%)
4. Isolated pulp horn remnants (20%)
5. No pulp horn (10%)

In this case report, the accessory cusp is situated centrally on the oblique ridge and the cuspal tip extends above the level of other cusps. Looking at the clinical presentation, it is assumed that the cause of formation of the central cusp is similar to dens evaginatus. S Nagarajan MP et al (2009)^15^ reported a case of accessory central cusps of the right and left maxillary deciduous second molars. He found that, the maxillary second primary molars when in occlusion, created depressions on the occlusal surface of both the opposing teeth (right and left mandibular 2nd deciduous molars). He also noted that, the cuspal tips of the accessory cusps showed some degree of attrition.

An interesting finding related to the central cusp in this case was presence of occlusal caries in both the maxillary and mandibular second molars without involving any other teeth. This phenomenon reveals a strong correlation of caries and central accessory cusp. The reason behind the caries affecting the maxillary second primary molars can be attributed to the food lodgement in the deep pits and fssures surrounding the central accessory cusps. Another factor can be the improper cuspal contacts between the maxillary and the mandibular second primary molars. The depressions caused by the maxillary molars were not evidenced in mandibular molars as they were grossly carious. A long-term observation is required until the permanent successors erupt; to know the evidence of dens evaginatus or the occurrence of any other morphological abnormalities.

## CONCLUSION

The presence of accessory cusps in this case-report was an incidental or rather accidental finding. Though it is reported as rare but we feel many of these cases either go unnoticed or report to the clinic in such a grossly decayed condition that these cusps get obliterated. Hence, careful monitoring of such cases is needed and early and prompt management should be implicated as soon as such findings are noted. The presence of these accessory cusps make very difficult to maintain routine oral healthcare in those sites. As the pits and grooves surrounding the cusps are highly susceptible to caries it is necessary to seal them at once. Premature contacts or occlusal interferences if present should be removed to prevent development of temperomandibular disorders or habitual posturing of the jaws. The anomalous teeth should be kept under periodic monitoring to check for caries status and pulp vitality.
